# Prognostic significance of WT1 expression in soft tissue sarcoma

**DOI:** 10.1186/1477-7819-12-214

**Published:** 2014-07-16

**Authors:** Ahrong Kim, Eun Young Park, Kyungbin Kim, Jeong Hee Lee, Dong Hoon Shin, Jee Yeon Kim, Do Youn Park, Chang Hun Lee, Mee Young Sol, Kyung Un Choi, Jeung Il Kim, In Sook Lee

**Affiliations:** 1 Department of Pathology, Pusan National University Hospital, Busan 602-739, Republic of Korea; 2 Department of Pathology, School of Medicine, Pusan National University, Yangsan, Gyeongsangnam-do 626-870, Republic of Korea; 3 Department of Pathology, Pusan National University Yangsan Hospital, Yangsan, Gyeongsangnam-do 626-770, Republic of Korea; 4 Department of Orthopedics, School of Medicine, Pusan National University, Yangsan, Gyeongsangnam-do 626-870, Republic of Korea; 5 Department of Radiology, School of Medicine, Pusan National University, Yangsan, Gyeongsangnam-do 626-870, Republic of Korea

## Abstract

**Background:**

Soft tissue sarcomas (STS) are rare. We evaluated the WT1 protein expression level in various types of STS and elucidated the value of WT1 as a prognostic factor and a possible therapeutic target.

**Methods:**

Immunohistochemical staining for WT1 was performed in 87 cases of STS using formalin-fixed, paraffin-embedded blocks. The correlation between WT1 expression and clinicopathological factors was analyzed. Survival analysis was conducted in 67 patients. We assessed the validity of WT1 immunohistochemistry as an index of WT1 protein expression using Western blot analysis.

**Results:**

WT1 expression was noted in 47 cases (54.0%). Most rhabdomyosarcomas and malignant peripheral nerve sheath tumors showed WT1 expression (91.7% and 71.4%, respectively; *P* = 0.005). WT1 expression was related to higher FNCLCC histologic grade and AJCC tumor stage. In the group with high grade STS, strong WT1 expression was correlated with better survival (*P* = 0.025). The immunohistochemical results were correlated quantitatively with the staining score and the concentration of the Western blot band.

**Conclusions:**

This study demonstrates that various types of STS show positive immunostaining for WT1 and that WT1 expression has a prognostic significance. So STS should be considered candidates for WT1 peptide--based immunotherapy.

## Background

The Wilms’ tumor gene (*WT1*) located at chromosome 11p13 was originally identified as a tumor-suppressor gene associated with Wilms’ tumor, a kidney neoplasm of childhood. The *WT1* is mutated in the germline of children with a genetic predisposition to Wilms’ tumor and is inactivated in a subset of sporadic Wilms’ tumors [1-3]. The *WT1* gene encodes a zinc-finger transcription factor, which regulates target genes, some of which are related to cell differentiation, proliferation, and apoptosis, and binds to specific sequences within the promoter regions of the *WT1* gene itself. It also binds to a number of other genes, such as *insulin like growth factor-II*, *platelet-derived growth factor A* chain, and *IGH-I* receptor [4-8]. The *WT1* gene also has a central role in embryonic development [9] and is normally expressed in a limited set of tissues, including gonad, uterus, kidney, and mesothelium [10-12].

Recent studies have suggested that *WT1* has an important role not only as a tumor suppressor, but also as a tumor promoter in various kinds of neoplasm. Many studies have shown that the wild-type *WT1* gene is expressed in leukemia [13,14], breast cancer [15,16], lung cancer [17], ovarian cancer [18], mesothelioma [19], renal cell carcinoma [20], and bone and soft tissue sarcomas [21,22]. However, the molecular pathway underlying the activity of *WT1* is still unclear. It is also not known whether the *WT1* gene is a tumor suppressor gene or an oncogene, or whether it has a biphasic function.

Many recent studies have highlighted the potential of the WT1 protein as a tumor-associated antigen and a candidate for targeted cancer immunotherapy. Clinical trials have suggested the safety and clinical efficacy of WT1 immunotherapy in cancer [23-25]. In 2007, the National Cancer Institute immunotherapy agent workshop [26] was held to rank agents with high potential to serve as immunotherapeutic drugs. According to its criteria, WT1 ranked the highest out of 75 cancer antigens prioritized. These results imply that a new era of WT1-targeted therapy is imminent.

Soft tissue sarcomas (STS) are rare malignant tumors accounting for about 1% of adult and 15% of pediatric malignancies [27]. *WT1* expression in STS, especially at the protein level, is not well documented. Ueda et al. [21] reported that various types of bone and soft tissue sarcomas frequently overexpress the wild-type *WT1* gene. They also reported that the *WT1* mRNA expression level can serve as a potent prognostic indicator in STS [22]. Nakatsuka et al. [28] reported that 70% of various types of STS expressed positive immunostaining for WT1.

In the current study, we evaluated the WT1 protein expression level by immunohistochemistry in various types of STS and assessed the validity of WT1 immunohistochemistry as an index of WT1 protein expression by comparison with Western blot analysis. In addition, we elucidated the value of *WT1* as a prognostic factor and the possibility of WT1 immunotherapy for STS.

## Methods

### Patients and tissue samples

STS samples were obtained from surgical operations carried out at Pusan National University Hospital, Korea, from 1998 to 2009. A total of 87 patients who underwent surgical resection for primary STS were included. Various clinicopathological data, including patient age, tumor size, metastasis at diagnosis, and details on the tumor grade and stage were obtained from the primary pathology reports and patient chart review. The histological diagnosis was determined by World Health Organization criteria, and the histological grade was determined according to the Federation Nationale des Centres de Lutte Contre le Cancer (FNCLCC) scheme. Surgical staging was determined based on the criteria recommended by the American Joint Committee on Cancer (AJCC). The aforementioned clinicopathological data were available for all 87 patients.

Fresh tumor tissue samples were used. The biospecimens for this study were provided by the Pusan National University Hospital, a member of the National Biobank of Korea, which is supported by the Ministry of Health, Welfare, and Family Affairs. All samples derived from the National Biobank of Korea were obtained with informed consent under institutional review board-approved protocols. The samples were snap-frozen in liquid nitrogen shortly after biopsy or resection and stored at −80°C until use. They included four cases of liposarcoma, one case of fibrosarcoma, one case of leiomyosarcoma, one case of malignant fibrous histiocytoma, and one case of synovial sarcoma. The results of analyses of the samples’ immunohistochemical expression and the Western blot were compared to validate the immunohistochemistry as an index of WT1 protein expression.

### Immunohistochemistry

Immunohistochemistry was performed on serial 4-μm thick paraffin sections. The paraffin sections were deparaffinized in xylene and rehydrated in a descending ethanol series. Bond Epitope Retrieval Solution 1 [pH ~ 6] or Bond Epitope Retrieval Solution 2 [pH ~ 9] (Leica Microsystems, Wetzlar, Germany) were used for antigen retrieval. Mouse monoclonal WT1 antibody (dilution 1:100, Clone 6 F-H2, Dako) was applied on the slides. Immunohistochemical staining was performed with a Leica Bond-MAX™ autostainer (Leica Microsystems, Berlin, Germany) and the peroxidase/DAB Bond™ Polymer Refine Detection System (Leica Microsystems) was used for visualization.

### Assessment of immunohistochemical staining

Evaluation of immunohistochemical staining was performed by two independent pathologists (Kim A and Choi KU). The stainings were scored while the pathologists were blinded to the clinicopathological data. WT1 was considered positive when cytoplasm and/or nuclear staining were observed [28]. The extent of expression was evaluated semi-quantitatively based on a staining score system after comparing the results of the immunostaining with those of RT-PCR [29]. The intensity of the staining and the proportion of the positive staining area were considered together. The intensity of the immunostaining was graded as 0 (negative), 1 (weak), 2 (moderate), or 3 (strong), and then the percentage of positive tumor cells was evaluated. The scoring system was based on the multiplication of the percentage and the intensity grade of positive cells, with the cells graded as negative (0–20), weak (21–80), moderate (81–180), or strong (181–300). Blood vessels, which open directly between tumor cells in sarcomas, were used as a positive control. Finally, to perform a statistical analysis, we grouped the four staining groups into two categories: negative (negative group) and positive expression (weak, moderate, and strong group).

### Western blot analysis

Proteins from the fresh frozen sarcoma tissue were loaded onto each well of the gel, separated by SDS-PAGE, and then transferred onto a membrane (CP-BU new, Agfa). After blocking nonspecific binding, the membrane was immunoblotted with the anti-WT1 mouse monoclonal antibody WLM 04 (Santa Cruz Biotechnology), followed by incubation with the appropriate secondary antibody conjugation with alkaline phosphatase.

### Statistical analysis

All statistical analyses were performed using SPSS for Windows software version 19.0 (SPSS Inc., Chicago, IL, USA). Pearson’s χ^2^ test was used to study the associations between the clinicopathological factors and WT1 expression. Overall survival (OS) was defined as the time being from the day of diagnosis until the death of the patient by Kaplan-Meier survival curves. Disease-free survival (DFS) was defined as the time from the day of diagnosis until any event including death, distant metastasis, or recurrence, by Kaplan-Meier survival curves. For all tests, a *P* value of less than 0.05 was considered significant. The survival analysis was performed between 1998 and 2007.

## Results

### Clinicopathological data

Patient age ranged from 1 to 82 years (median age 50 years) and there were 49 males and 38 females. There were 26 cases of liposarcoma, 21 cases of malignant fibrous histiocytoma, 12 cases of rhabdomyosarcoma, 6 cases of leiomyosarcoma, 7 cases of malignant peripheral nerve sheath tumor (MPNST) and synovial sarcoma, 5 cases of fibrosarcoma, and 3 cases of other sarcomas including epithelioid sarcoma and alveolar soft part sarcoma. Three patients had metastatic sarcoma upon initial diagnosis. Other detailed clinicopathological data are shown in Table 1.

**Table 1:** Clinicopathological features (n = 87)

**Clinicopathologic data**	**Cases**
Age (median, mean)	50, 49
Age	
Less than 50 yrs	40
More than 50 yrs	47
Sex	
Male	49
Female	38
Histologic type	
Liposarcoma	26
MFH	21
Rhabdomyosarcoma	12
Leiomyosarcoma	6
MPNST	7
Synovial sarcoma	7
Fibrosarcoma	5
Others*	3
Site	
Thigh	27
Upper arm	5
Retroperitoneum	8
Head and neck	11
Buttock	5
Forearm	5
Back	3
Lower leg	4
Others	19
Tumor size	
<5 cm	13
>5 cm	74
FNCLCC grade	
1	17
2	37
3	33
AJCC stage	
1	17
2	42
3	25
4	3
Total	87

*Others include 2 alveolar soft part sarcomas and 1 epithelioid sarcoma.

MFH, Malignant fibrous histiocytoma; MPNST, Malignant peripheral nerve sheath tumor; FNCLCC, Federation Nationale des Centres de Lutte Contre le Cancer; AJCC, American Joint Committee on Cancer.

### WT1 immunohistochemistry

**Figure 1:**
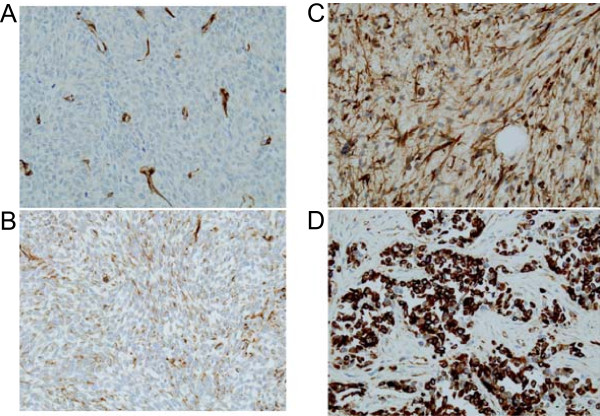
**Immunohistochemical staining of WT1 (×400). (A)** Negative staining. Note that this section shows positive staining for blood vessels. **(B)** Weak staining. **(C)** Moderate staining. **(D)** Strong staining.

A total of 87 STS samples were used for the determination of WT1 expression. All of the samples showed positive staining for feeding blood vessels, demonstrating that the staining was reliable and appropriate (Figure 1). Specific staining for WT1 was observed in the cytoplasm of tumor cells, but in a few cases, both cytoplasmic and nuclear staining was observed. There was negative staining in 40 specimens (negative, 46.0%). The staining for WT1 showed weak positivity in 6 (6.9%), moderate positivity in 15 (17.2%), and strong positivity in 26 (29.9%). Thus, WT1 expression was noted in 47 (54.0%) of the 87 cases by immunohistochemistry.

### Correlation between WT1 expression level and clinicopathological characteristics of STS

Table 2 summarizes the association of WT1 expression in STS with the clinicopathological parameters. WT1 expression was associated with the histological diagnosis. Rhabdomyosarcoma was strongly correlated with WT1 expression (91.7%, *P* = 0.005). WT1 expression was associated with high FNCLCC grade and advanced tumor stage (*P* = 0.000).

**Table 2:** Correlation between WT1 expression and clinicopathological factors

**Clinicopathologic parameters**	**WT-1 protein expression**		
	**Absent, n = 40 (46.0%)**	**Present, n = 47 (54.0%)**	***P *** **value**
Age			
<50	19 (47.5)	21 (52.5)	0.793
>50	21 (44.7)	26 (55.3)	
Histologic type			
Liposarcoma	20 (76.9)	6 (23.1)	0.005
MFH	9 (42.9)	12 (57.1)	
Leiomyosarcoma	2 (33.3)	4 (66.7)	
Rhabdomyosarcoma	1 (8.3)	11 (91.7)	
MPNST	2 (28.6)	5 (71.4)	
Synovial sarcoma	3 (42.9)	4 (57.1)	
Fibrosarcoma	1 (20.0)	4 (80.0)	
Others	2 (40.0)	3 (60.0)	
Size			
<5 cm	3 (23.1)	10 (76.9)	0.072
>5 cm	37 (50.0)	37 (50.0)	
Metastasis at Dx			
Absent	38 (45.8)	45 (54.2)	0.869
Present	2 (50.0)	2 (50.0)	
FNCLCC grade			
1	16 (94.1)	1 (5.9)	0.000
2	14 (37.8)	23 (62.2)	
3	10 (30.3)	23 (69.7)	
Modified grade *			
Low	16 (94.1)	1 (5.9)	0.000
High	24 (34.3)	46 (65.7)	
AJCC stage			
1	15 (83.3)	3 (16.7)	0.003
2	14 (34.1)	27 (65.9)	
3	9 (36.0)	16 (64.0)	
4	2 (66.7)	1 (33.3)	
AJCC modified**			
Localized	15 (83.3)	3 (16.7)	0.000
Advanced	25 (36.2)	44 (63.8)	

*FNCLCC grade was divided into 2 groups, low grade including FNCLCC group 1 and high grade including FNCLCC grade 2 and 3.

**AJCC stage 1 was defined as localized disease and AJCC stage 2, 3, and 4 were defined as advanced disease.

MFH, Malignant fibrous histiocytoma; MPNST, Malignant peripheral nerve sheath tumor; FNCLCC, Federation Nationale des Centres de Lutte Contre le Cancer; AJCC, American Joint Committee on Cancer.

### Correlation between WT1 expression and survival

Clinical follow-up data were available for 63 patients. The median follow-up was 29 months (1–187). Twenty-seven patients developed local recurrence, and 17 patients developed metastasis; 25 patients (39.6% of total patients) died of the disease during the follow-up period.

In the group for which follow-up data were available (n = 63), 15 (45.4%) of 33 patients with WT1 expression died of the disease, compared to 10 (33.3%) of 30 patients without WT1 expression; this difference was not statistically significant (*P* = 0.326). Twenty-three (69.6%) of 33 patients with WT1 expression had disease-related events, including recurrence, distant metastasis, and death, compared to 18 (60.0%) of 30 patients without WT1 expression; this difference was not statistically significant (*P* = 0.420) (Table 3).

**Table 3:** Correlation between WT1 expression and survival

	**WT-1 protein expression**		
	**Absent, n = 30 (47.6%)**	**Present, n = 33 (52.4%)**	***P *** **value**
Survival*			
Survival	20 (52.6)	18 (47.4)	0.326
Death	10 (40.0)	15 (60.0)	
Any event*,**			
Absent	12 (54.5)	10 (45.5)	0.420
Present	18 (43.9)	23 (56.1)	

*Evaluation for survival and any event was performed among patients between 1998 and 2007.

**Any events include local recurrence, distant metastasis, and death during follow-up.

**Figure 2:**
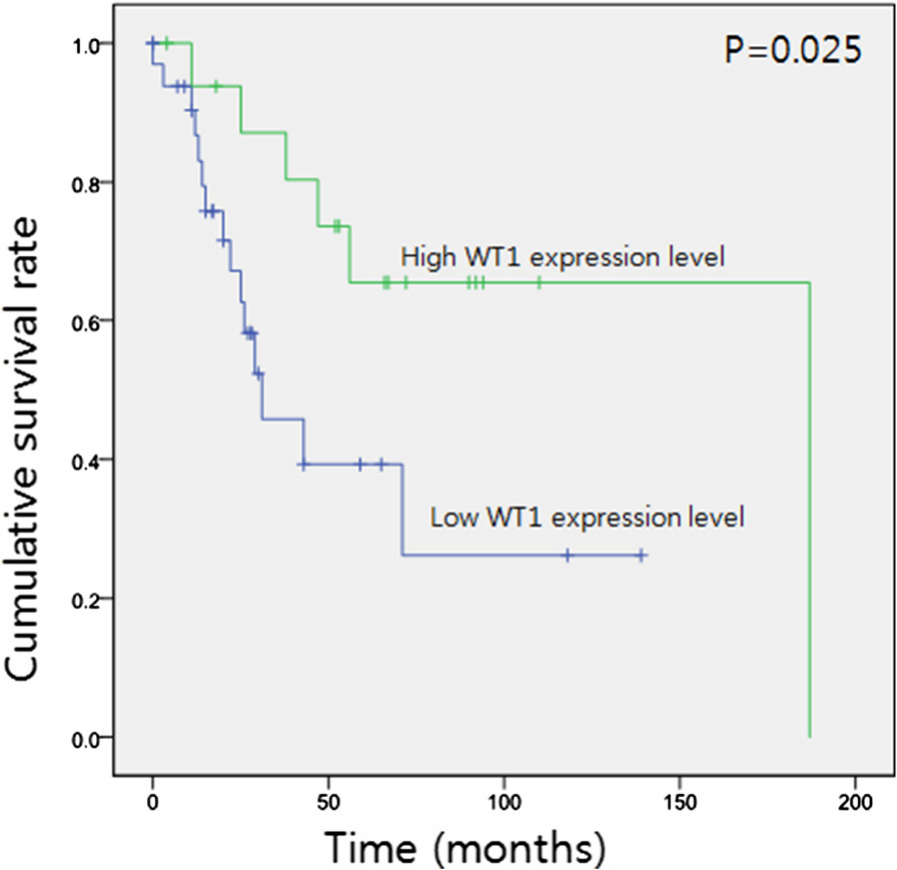
**Overall survival of high grade soft tissue sarcomas in low WT1 expression level and high WT1 expression level (*P*** **= 0.025).**

In the survival analysis of the group with high grade STS (n = 50), WT1 expression was not correlated with OS and DFS (*P* = 0.710, *P* = 0.728, respectively). However, 6 (35.2%) of 17 patients with strong WT1 expression and 15 (45.4%) of 33 patients in a remnant group (including negative, weak, and moderate expression) died of the disease. Strong WT1 expression was associated with a better outcome in the group with high grade sarcoma (*P* = 0.025) (Figure 2).

### Western blot analysis

**Figure 3:**
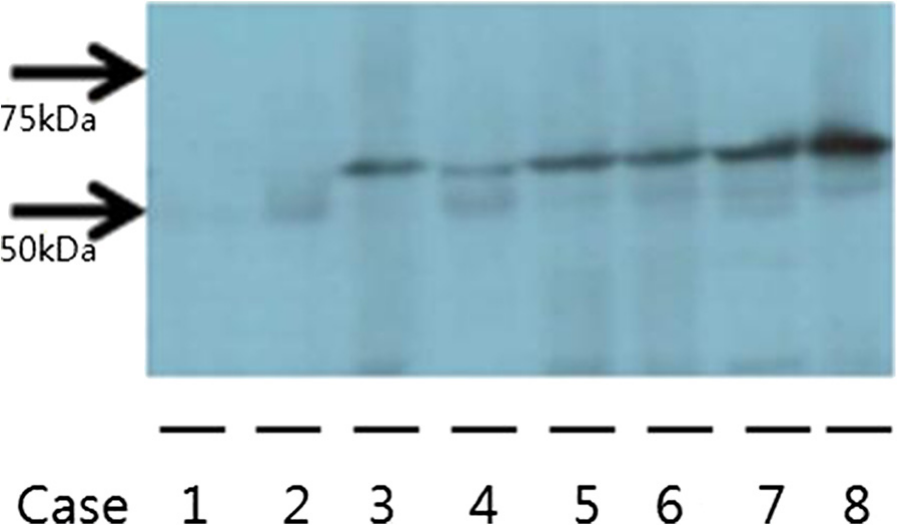
**Western blot analysis revealed that immunohistochemical expression of WT1 is a precise method of evaluation for WT1 protein expression, compared with counterpart immunohistochemical staining (see Table**3**).**

To determine the accuracy of the immunohistochemical staining as an index of protein expression, the results of the Western blot analysis were compared with those of the immunohistochemical staining. The Western blot analysis revealed not only that immunohistochemical staining is a reliable method for the evaluation of protein expression but also that the immunohistochemistry correlates quantitatively with the staining score and the concentration of the Western blot band (Table 4 and Figure 3).

**Table 4:** WT1 expression by immunohistochemistry for comparison with western blot

	**WT-1 protein expression**			
	**Intensity**	**% of positive cells**	**Score**	
Case 1 Liposarcoma	1	10	10	Negative
Case 2 Liposarcoma	1	20	20	Negative
Case 3 Synovical sarcoma	1	40	40	Positive
Case 4 Liposarcoma	2	20	40	Positive
Case 5 Malignant fibrous histiocytoma	2	20	40	Positive
Case 6 Liposarcoma	1	45	45	Positive
Case 7 Fibrosarcoma	2	40	80	Positive
Case 8 Leiomyosarcoma	2	95	190	Positive

## Discussion

In this study, we investigated WT-1 expression at the protein level by immunohistochemistry in various kinds of STS and examined the correlation between the WT1 expression level and clinicopathological factors.

The study of WT1 in STS is limited. To our knowledge, this is the largest cohort study of WT1 expression in STS to date. There also appears to have been no report about the relationship between WT1 expression at the protein level by immunohistochemistry and prognosis.

More than half (54.0%) of STS showed positive expression for WT1 immunostaining, demonstrating that more than half of STS are candidates for WT1-targeted immunotherapy. Nakatsuka et al. [28] reported that 70% of 32 soft tissue sarcomas showed positivity for WT1 immunostaining and that 100% of rhabdomyosarcomas, malignant fibrous histiocytomas, MPNSTs, and clear cell sarcomas showed positive staining for WT1 when using monoclonal (6 F-H2) antibody. The higher frequency of WT1 positivity in their study was likely due to the fact that they considered only the positivity of staining, while we considered not only the positivity, but also the intensity and the proportion of positivity. After multiplying the percentage and the intensity grade of the positive cells, a score between 0 and 20 was considered negative in the present study. Our results revealed that rhabdomyosarcoma and MPNST showed frequent WT1 expression (91.7% and 71.4%, respectively). It seems reasonable that WT1 cancer immunotherapy should be considered, especially in rhabdomyosarcoma and MPNST.

Ueda et al. reported that the *WT1* gene was frequently overexpressed in various types of STS [21] and that *WT1* mRNA overexpression was significantly associated with a poor prognosis. However, the current study did not reveal any association between WT1 expression and OS or DFS. The results for OS and DFS among high grade STS were similar. It is interesting that strong WT1 expression was correlated with better survival in the group of high grade STS. Only 2 out of 10 cases of high grade rhabdomyosarcoma with strong WT1 expression died as a result of the disease, while all two patients of high grade rhabdomyosarcoma with weak WT1 expression died of disease. Although Ueda et al. determined that the WT1 mRNA level was correlated with the WT1 protein level by immunoblotting [22] and immunohistochemistry [21], this was the case in only 4 of 52 and 3 out of 36 samples, respectively. Therefore, the correlation between the protein and mRNA level of the *WT1* gene has not been conclusively validated. Based on the results of the comparison of the immunostaining with those of the western blot analysis, the current study demonstrates that WT1 immunohistochemical staining is a reliable method for evaluating the WT1 protein expression level. As previously mentioned, it is not known whether the *WT1* gene is a tumor suppressor or an oncogene, or whether it has a biphasic function. The molecular pathway also remains to be further defined. Therefore, further studies on the correlation between the protein and mRNA of the *WT1* gene in larger cohort are required, together with survival analysis, to validate of the WT1 expression level as a prognostic factor.

High expression of the *WT1* gene in solid cancers and leukemia suggested that the WT1 protein might be a possible tumor-associated antigen. In a mouse model, murine WT1 protein-derived, MHC class I-restricted WT1 peptides were tested to induce WT1-specific cytotoxic T lymphocytes (CTLs). The WT1-specific CTLs in mice lysed WT1-expressing tumor cells showed no evidence of histopathological damage of organs that physiologically expressed WT1 [23,30,31]. The mechanism by which WT1-specific CTLs ignore WT1-expressing normal cells is not clear, but there are four probable mechanisms. First, it may be due to normal cells having lower WT1 expression than tumor cells. However, this is unlikely considering that the level of WT1 expression in CD34+ normal hematopoietic progenitor cells is as high as in leukemic cells. Second, the expression of MHC class I molecules may be lower in physiologically WT1 expressing normal cells than in WT1 expressing tumor cells. Third, the WT1 peptide presentation of WT1-expressing normal cells may be poor. Fourth, WT1-expressing normal cells do not express, or weakly express, cell surface costimulatory molecules required for recognition and/or killing by WT1-specific CTLs [24].

WT1 peptide immunotherapy has also been confirmed to have the clinical effectiveness and safety in the phase I study with solid tumors or hematopoietic malignancies [32]. Localized skin erythema at the injected site was the only adverse effect in patients with normal hematopoiesis [24,33]. Ohta el al. [34] reported that WT1 peptide vaccination was effective in a pediatric patient with metastatic alveolar rhabdomyosarcoma who showed poor response to chemotherapy, and the patient had no adverse effects other than skin erythema.

For STS, surgery alone or in combination with radiotherapy and chemotherapy is the mainstream treatment, and the survival rate has changed just little in recent decades [35]. However, chemotherapy and radiotherapy usually have significant systemic side effects and it is well documented that radiation itself is a risk factor of cancer. WT1 peptide immunotherapy has no significant side effects other than localized skin erythema and is more promising in aspect that soft tissue sarcomas are more common in childhood.

Recently, 75 representative cancer antigens were prioritized according to the following criteria: i) therapeutic function, ii) immunogenicity, iii) role of the antigen in oncogenicity, iv) specificity, v) expression level and percentage of antigen positive cells, vi) stem cell expression, vii) number of patients with antigen positive cancers, viii) number of antigenic epitopes, and ix) cellular location of antigen expression [26]. They reported that WT1 was at the top of the ranking. WT1 peptide-based immunotherapy will be a routine option for malignant tumor treatment in the near future. Our study suggests that STS patients are appropriate candidates for WT1 immunotherapy.

This study demonstrates that various types of STS shows positive cytoplasmic immunostaining for WT1 and STS patients should be considered candidates for WT1 peptide-based immunotherapy, particularly in cases of rhabdomyosarcoma and MPNST. To validate the role of WT1 as a prognostic factor, further studies for molecular pathways and survival analyses in larger cohorts would be helpful.

## Conclusions

Our study revealed that WT-1 was expressed in the cytoplasm of the tumor cells of a large number of STS using immunohistochemistry. Rhabdomyosarcomas and MPNST showed WT1 expression in a high proportion. WT1 expression was related to higher FNCLCC histologic grade and AJCC tumor stage. In the group with high grade STS, strong WT1 expression was correlated with better survival. The immunohistochemical results were correlated with those of the Western blot. Our data indicated that the cytoplasmic WT1 expression may have prognostic significance in high grade STS and various kinds of soft tissue sarcomas are candidates for WT1 targeted immunotherapy.

## Abbreviations

CTLs: Cytotoxic T lymphocytes; DFS: Disease-free survival; FNCLCC: Federation Nationale des Centres de Lutte Contre le Cancer; MFH: Malignant fibrous histiocytoma; MPNST: Malignant peripheral nerve sheath tumor; OS: Overall survival; STS: Soft tissue sarcomas; *WT1*: Wilms’ tumor gene.

## Competing interests

The authors declare that they have no competing interests.

## Authors’ contributions

AK and EYP collected data, performed analysis, and drafted, revised, and finalized the manuscript. KUC conceived this study and participated in its design and coordination. KK, JHL, DHS, JYK, DYP, CHL, and MYS performed analysis, and revised and approved the contents of the manuscript. All authors read and approved the final manuscript.

## References

[B1] Call KM Glaser T Ito CY Buckler AJ Pelletier J Haber DA Rose EA Kral A Yeger H Lewis WH Isolation and characterization of a zinc finger polypeptide gene at the human chromosome 11 Wilms’ tumor locus Cell 1990 60 509 520 2154335 10.1016/0092-8674(90)90601-a

[B2] Gessler M Poustka A Cavenee W Neve RL Orkin SH Bruns GA Homozygous deletion in Wilms tumours of a zinc-finger gene identified by chromosome jumping Nature 1990 343 774 778 2154702 10.1038/343774a0

[B3] Pelletier J Bruening W Li FP Haber DA Glaser T Housman DE WT1 mutations contribute to abnormal genital system development and hereditary Wilms’ tumour Nature 1991 353 431 434 1654525 10.1038/353431a0

[B4] Scharnhorst V van der Eb AJ Jochemsen AG WT1 proteins: functions in growth and differentiation Gene 2001 273 141 161 11595161 10.1016/s0378-1119(01)00593-5

[B5] Reddy JC Licht JD The WT1 Wilms’ tumor suppressor gene: how much do we really know? Biochim Biophys Acta 1996 1287 1 28 8639704 10.1016/0304-419x(95)00014-7

[B6] Drummond IA Madden SL Rohwer-Nutter P Bell GI Sukhatme VP Rauscher FJ 3rd Repression of the insulin-like growth factor II gene by the Wilms tumor suppressor WT1 Science 1992 257 674 678 1323141 10.1126/science.1323141

[B7] Wang ZY Madden SL Deuel TF Rauscher FJ 3rd The Wilms’ tumor gene product, WT1, represses transcription of the platelet-derived growth factor A-chain gene J Biol Chem 1992 267 21999 22002 1429549

[B8] Werner H Re GG Drummond IA Sukhatme VP Rauscher FJ 3rd Sens DA Garvin AJ LeRoith D Roberts CT Jr Increased expression of the insulin-like growth factor I receptor gene, IGF1R, in Wilms tumor is correlated with modulation of IGF1R promoter activity by the WT1 Wilms tumor gene product Proc Natl Acad Sci U S A 1993 90 5828 5832 8390684 10.1073/pnas.90.12.5828 PMC46816

[B9] Coosemans A Van Calster B Verbist G Moerman P Vergote I Van Gool SW Amant F Wilms tumor gene 1 (WT1) is a prognostic marker in high-grade uterine sarcoma Int J Gynecol Cancer 2011 21 302 308 21734473 10.1097/IGC.0b013e318207cab5

[B10] Buckler AJ Pelletier J Haber DA Glaser T Housman DE Isolation, characterization, and expression of the murine Wilms’ tumor gene (WT1) during kidney development Mol Cell Biol 1991 11 1707 1712 1671709 10.1128/mcb.11.3.1707-1712.1991 PMC369476

[B11] Park S Schalling M Bernard A Maheswaran S Shipley GC Roberts D Fletcher J Shipman R Rheinwald J Demetri G The Wilms tumour gene WT1 is expressed in murine mesoderm-derived tissues and mutated in a human mesothelioma Nat Genet 1993 4 415 420 8401592 10.1038/ng0893-415

[B12] Davies R Moore A Schedl A Bratt E Miyahawa K Ladomery M Miles C Menke A van Heyningen V Hastie N Multiple roles for the Wilms’ tumor suppressor, WT1 Cancer Res 1999 59 1747s 1750s 10197591

[B13] Inoue K Ogawa H Sonoda Y Kimura T Sakabe H Oka Y Miyake S Tamaki H Oji Y Yamagami T Tatekawa T Soma T Kishimoto T Sugiyama H Aberrant overexpression of the Wilms tumor gene (WT1) in human leukemia Blood 1997 89 1405 1412 9028964

[B14] Miwa H Beran M Saunders GF Expression of the Wilms’ tumor gene (WT1) in human leukemias Leukemia 1992 6 405 409 1317488

[B15] Loeb DM Evron E Patel CB Sharma PM Niranjan B Buluwela L Weitzman SA Korz D Sukumar S Wilms’ tumor suppressor gene (WT1) is expressed in primary breast tumors despite tumor-specific promoter methylation Cancer Res 2001 61 921 925 11221883

[B16] Silberstein GB Van Horn K Strickland P Roberts CT Jr Daniel CW Altered expression of the WT1 wilms tumor suppressor gene in human breast cancer Proc Natl Acad Sci U S A 1997 94 8132 8137 9223327 10.1073/pnas.94.15.8132 PMC21569

[B17] Oji Y Miyoshi S Maeda H Hayashi S Tamaki H Nakatsuka S Yao M Takahashi E Nakano Y Hirabayashi H Shintani Y Oka Y Tsuboi A Hosen N Asada M Fujioka T Murakami M Kanato K Motomura M Kim EH Kawakami M Ikegame K Ogawa H Aozasa K Kawase I Sugiyama H Overexpression of the Wilms’ tumor gene WT1 in de novo lung cancers Int J Cancer 2002 100 297 303 12115544 10.1002/ijc.10476

[B18] Bruening W Gros P Sato T Stanimir J Nakamura Y Housman D Pelletier J Analysis of the 11p13 Wilms’ tumor suppressor gene (WT1) in ovarian tumors Cancer Invest 1993 11 393 399 8324644 10.3109/07357909309018871

[B19] Amin KM Litzky LA Smythe WR Mooney AM Morris JM Mews DJ Pass HI Kari C Rodeck U Rauscher FJ 3rd Wilms’ tumor 1 susceptibility (WT1) gene products are selectively expressed in malignant mesothelioma Am J Pathol 1995 146 344 356 7856747 PMC1869867

[B20] Campbell CE Kuriyan NP Rackley RR Caulfield MJ Tubbs R Finke J Williams BR Constitutive expression of the Wilms tumor suppressor gene (WT1) in renal cell carcinoma Int J Cancer 1998 78 182 188 9754650 10.1002/(sici)1097-0215(19981005)78:2<182::aid-ijc11>3.0.co;2-d

[B21] Ueda T Oji Y Naka N Nakano Y Takahashi E Koga S Asada M Ikeba A Nakatsuka S Abeno S Hosen N Tomita Y Aozasa K Tamai N Myoui A Yoshikawa H Sugiyama H Overexpression of the Wilms’ tumor gene WT1 in human bone and soft-tissue sarcomas Cancer Sci 2003 94 271 276 12824921 10.1111/j.1349-7006.2003.tb01432.x PMC11160304

[B22] Sotobori T Ueda T Oji Y Naka N Araki N Myoui A Sugiyama H Yoshikawa H Prognostic significance of Wilms tumor gene (WT1) mRNA expression in soft tissue sarcoma Cancer 2006 106 2233 2240 16607650 10.1002/cncr.21861

[B23] Oka Y Udaka K Tsuboi A Elisseeva OA Ogawa H Aozasa K Kishimoto T Sugiyama H Cancer immunotherapy targeting Wilms’ tumor gene WT1 product J Immunol 2000 164 1873 1880 10657636 10.4049/jimmunol.164.4.1873

[B24] Oka Y Tsuboi A Taguchi T Osaki T Kyo T Nakajima H Elisseeva OA Oji Y Kawakami M Ikegame K Hosen N Yoshihara S Wu F Fujiki F Murakami M Masuda T Nishida S Shirakata T Nakatsuka S Sasaki A Udaka K Dohy H Aozasa K Noguchi S Kawase I Sugiyama H Induction of WT1 (Wilms’ tumor gene)-specific cytotoxic T lymphocytes by WT1 peptide vaccine and the resultant cancer regression Proc Natl Acad Sci U S A 2004 101 13885 13890 15365188 10.1073/pnas.0405884101 PMC518848

[B25] Ohno S Kyo S Myojo S Dohi S Ishizaki J Miyamoto K Morita S Sakamoto J Enomoto T Kimura T Oka Y Tsuboi A Sugiyama H Inoue M Wilms’ tumor 1 (WT1) peptide immunotherapy for gynecological malignancy Anticancer Res 2009 29 4779 4784 20032435

[B26] Cheever MA Allison JP Ferris AS Finn OJ Hastings BM Hecht TT Mellman I Prindiville SA Viner JL Weiner LM Matrisian LM The prioritization of cancer antigens: a national cancer institute pilot project for the acceleration of translational research Clin Cancer Res 2009 15 5323 5337 19723653 10.1158/1078-0432.CCR-09-0737 PMC5779623

[B27] Mazanet R Antman KH Sarcomas of soft tissue and bone Cancer 1991 68 463 473 2065265 10.1002/1097-0142(19910801)68:3<463::aid-cncr2820680304>3.0.co;2-e

[B28] Nakatsuka S Oji Y Horiuchi T Kanda T Kitagawa M Takeuchi T Kawano K Kuwae Y Yamauchi A Okumura M Kitamura Y Oka Y Kawase I Sugiyama H Aozasa K Immunohistochemical detection of WT1 protein in a variety of cancer cells Mod Pathol 2006 19 804 814 16547468 10.1038/modpathol.3800588

[B29] Coosemans A Nik SA Caluwaerts S Lambin S Verbist G Van Bree R Schelfhout V de Jonge E Dalle I Jacomen G Cassiman JJ Moerman P Vergote I Amant F Upregulation of Wilms’ tumour gene 1 (WT1) in uterine sarcomas Eur J Cancer 2007 43 1630 1637 17531467 10.1016/j.ejca.2007.04.008

[B30] Tsuboi A Oka Y Ogawa H Elisseeva OA Li H Kawasaki K Aozasa K Kishimoto T Udaka K Sugiyama H Cytotoxic T-lymphocyte responses elicited to Wilms’ tumor gene WT1 product by DNA vaccination J Clin Immunol 2000 20 195 202 10941827 10.1023/a:1006637529995

[B31] Gaiger A Reese V Disis ML Cheever MA Immunity to WT1 in the animal model and in patients with acute myeloid leukemia Blood 2000 96 1480 1489 10942395

[B32] Morita S Oka Y Tsuboi A Kawakami M Maruno M Izumoto S Osaki T Taguchi T Ueda T Myoui A Nishida S Shirakata T Ohno S Oji Y Aozasa K Hatazawa J Udaka K Yoshikawa H Yoshimine T Noguchi S Kawase I Nakatsuka S Sugiyama H Sakamoto J A phase I/II trial of a WT1 (Wilms’ tumor gene) peptide vaccine in patients with solid malignancy: safety assessment based on the phase I data Jpn J Clin Oncol 2006 36 231 236 16611662 10.1093/jjco/hyl005

[B33] Oka Y Tsuboi A Murakami M Hirai M Tominaga N Nakajima H Elisseeva OA Masuda T Nakano A Kawakami M Oji Y Ikegame K Hosen N Udaka K Yasukawa M Ogawa H Kawase I Sugiyama H Wilms tumor gene peptide-based immunotherapy for patients with overt leukemia from myelodysplastic syndrome (MDS) or MDS with myelofibrosis Int J Hematol 2003 78 56 61 12894852 10.1007/BF02983241

[B34] Ohta H Hashii Y Yoneda A Takizawa S Kusuki S Tokimasa S Fukuzawa M Tsuboi A Murao A Oka Y Oji Y Aozasa K Nakatsuka S Sugiyama H Ozono K WT1 (Wilms tumor 1) peptide immunotherapy for childhood rhabdomyosarcoma: a case report Pediatr Hematol Oncol 2009 26 74 83 19206012 10.1080/08880010802435500

[B35] Weitz J Antonescu CR Brennan MF Localized extremity soft tissue sarcoma: improved knowledge with unchanged survival over time J Clin Oncol 2003 21 2719 2725 12860950 10.1200/JCO.2003.02.026

